# Assessing the Validity of a Physical Activity Questionnaire Developed for Parents of Preschool Children in Mexico

**DOI:** 10.3329/jhpn.v30i4.13327

**Published:** 2012-12

**Authors:** Montserrat Bacardi-Gascón, Claudia Reveles-Rojas, Gail Woodward-Lopez, Patricia Crawford, Arturo Jiménez-Cruz

**Affiliations:** ^1^Facultad de Medicina y Psicología, Universidad Autónoma de Baja California, México, Unidad Universitaria, Avda, Universidad 14418, Parque Industrial Internacional, 22390 Tijuana, B.C., Mexico;; ^2^Center for Weight and Health, University of California-Berkeley, 3 Giannini Hall, Berkeley, CA 94720, USA

**Keywords:** Accelerometer, Physical activity, Preschool children, Validity assessment, Mexico

## Abstract

To assess the validity of a questionnaire developed for parents of preschool children to know their physical activity (PA) status, we compared the questionnaire results with the measures of accelerometer for children's activities. Thirty-five preschoolers who wore the accelerometer for at least 10 hours daily on 3 weekdays and one weekend day were included in the analyses. Time spent in activities of varied intensity was calculated by applying 15-second ActiGraph count cutoffs (ACC). Parents’ perceptions of their children's PA were associated with the percentage of vigorous and moderate physical activity recorded with ACC at r=0.62 (p=0.0001). An association was shown between the percentage of a child's time spent in vigorous physical activity, as reported by parents, with that measured by ACC at r=0.53 (p=0.001). Results of this study suggest that the designed questionnaire might be a useful tool for assessing children's activity while, additionally, it warrants further investigation on larger samples of children.

## INTRODUCTION

The reported prevalence of overweight and obesity among five-year old children in Mexico is 17.7% (1), and among 3-5 year old children in poor, rural Mexico, the rate is greater than 23% (2). The preschool years are identified as a period of risk for the development of obesity that may last up to adulthood (3,4). Several authors have pointed out that decreased physical activity (PA) or high levels of sedentary behaviour are contributing to an increase in childhood overweight (5,6,7,8), and that preschoolers do not meet PA recommendations (9,10).

To obtain survey data on young children's PA, it is necessary to rely on reports from parents or adult caregivers since young children are unable to accurately self-report their physical activities (11). Sets of questionnaire administered to caregivers to assess young children's usual or actual PA have added benefits: these are inexpensive, non-invasive, and less time-consuming to administer and interpret (12). Given these benefits, careful evaluation of the validity of reports based on questionnaire is merited (13).

Accelerometer is considered to be a reliable instrument for the measurement of PA in preschool children and, therefore, are used for validation of other measures, including questionnaire (14,15,16,17). Further, sedentary behaviour can be quantified using accelerometry in preschool children (18). Since children engage in very short bursts of intense PA, along with varying intervals of low and moderate intensity (19,11,20), the use of the accelerometer set at 15 seconds (15-s) increment is recommended. Several studies have used accelerometers to evaluate the validity of different types of questionnaire used for parents to assess preschoolers’ PA (21,22,23,24). Harro used a daily registry of activities during working-days (21); Chen *et al.* assessed the frequencies and preferences of PA, using a questionnaire for teachers (22); Burdette *et al.* used a checklist to assess outdoor playing (23); and Janz *et al.* assessed preferences and choices of everyday activity (24). The ActiGraph uniaxial accelerometer has been evaluated and calibrated using 15-s data-collection increment with direct observation (15) and using a metabolic criterion measure (VO2) (25) for 3-5 year old children. However, we have found no simple tools to assess usual PA among preschool children. Little is known about the PA behaviour of preschool children in Mexico, and no questionnaire has been developed and validated for assessment of PA in Mexican preschool children. Parental perception of children's PA and the degree of understanding of the questionnaire might be determined by cultural patterns; therefore, translating a questionnaire developed for parents with different levels of education might yield inaccurate results. Designing of the culturally-appropriate questionnaire is warranted. Besides, due to the high percentage of the Mexican population with low reading and comprehension levels, a simpler tool for assessing usual PA needs to be evaluated. Thus, the aim of this study was to assess the validity of a physical activity questionnaire developed for use by parents of Mexican preschool children.

## MATERIALS AND METHODS

### Participants

Forty-five 3-5 year old children enrolled in 3 classes in a preschool care centre in Tijuana, Mexico and their parents were recruited to participate in this cross-sectional study. Children attended the preschool care centre four hours daily, from 9:00 am to 1:00 pm, Monday through Friday. The study protocol and procedures for obtaining the children's assent and informed consent of their parents were reviewed and approved by the Committee for the Protection of Human Subjects, University of California at Berkeley and the Committee for the Protection of Human Subjects, Medical School of Universidad Autónoma de Baja California (26).

### Settings

Tijuana city is at the extreme northwestern part of Mexico, and it borders the state of California, USA. In 2006, Tijuana had approximately 1,795,000 residents, and approximately 9% of the population was younger than 5 years. Government's projections indicate that 77% of preschool children were enrolled at care centres (27).

### Anthropometric measurements

Height was measured to the nearest millimetre, with a portable stadiometer (model 214 Seca Corp., Hanover, MD, USA), and weight was measured with electronic scales (model Tanita UM-028, Tokyo, Japan) to the nearest 0.1 kg. Body mass index (BMI) was calculated in kg/m^2^. BMI values were compared with age/gender BMI percentiles from the Centers for Disease Control and Prevention Growth Charts (28).

### Accelerometer data

PA data on 45 children were quantified based on ActiGraph GT1M accelerometer (ActiGraph, Fort Walton Beach, FL) data collected during weekdays (home and preschool centre) and weekends (home) between October 2006 and March 2007. Only 35 participants (3-5 years of age) (51% girls) who wore the accelerometer on the right hip for at least 10 hours daily on 3 weekdays and one weekend day were included in the analyses. Ten children who did not meet the adequate daily hours on 3 weekdays and one weekend as recommended were excluded. The ActiGraph was programmed to record activity counts in 15-second intervals to detect time spent in spontaneous activities. Children wore the accelerometers on the right hip, secured with an elastic belt. Parents and preschool teachers were instructed by a researcher on how to place and monitor the ActiGraph correctly during the day (CR). Parents placed the accelerometers on their children each morning and removed those at night prior to bedtime. Activity counts for each 15-s interval were obtained from the Actilife GTM1 software (version 2.1.9) developed to process the data from the ActiGraph GTM1. Time spent in PA of different intensities at preschool centre and at home was calculated by applying two different 15-s ActiGraph count cutoffs (ACC) that represent two different methods of deriving cutoff points as follows:

- The age-specific cutoff typology developed by Sirard *et al.* based on age-dependent ROC curves of the age-specific counts/15-s cutoffs for 3, 4, and 5 years for the activities of different intensities (15): sedentary (0–301), (0–363), and (0–398); light (302–614), (364–811), and (399–890); moderate (615–1230), (812–1234), and (891–1254); and vigorous (≥1231), (≥1235), and (≥1255). The ACC for PA of moderate and vigorous intensity were set at ≥615, ≥812, and ≥891 counts/15-s.
- The cutoff typology developed by Pate *et al.* was based on measuring VO_2_ while children performed unstructured PA (25). The ACC for PA of moderate and vigorous intensity was set at 420 counts/15-s (VO_2_=20 mL/kg) and 842 counts/15-s (VO_2_=30 mL/kg) respectively.

Two different data files were made—one with data collected during school hours and another including data collected during weekdays and weekends.

### Questionnaire

A six-item questionnaire was developed for and completed by parents (Appendix A) on the days when the children wore the accelerometers. The questionnaire was piloted for comprehension and reproducibility. Twenty-one parents participated in the pilot study. The questionnaire was self-administered, and the research assistant clarified any questions about its application. Six questions asked parents to report the usual amount of time per day spent in sleeping, naps, and various indoor and outdoor activities, which were grouped according to PA intensity (sedentary, moderate, and vigorous). From the information provided by parents through the questionnaire, the amount of daily time spent in different intensities of PA was computed in minutes and as a percentage of time at home and preschool centre. Sports and supervised activities were recorded weekly and computed to minutes/day of different PA levels according to the reported activity.

One additional question (Appendix) was asked to assess parents’ overall perception of the child's typical PA level by assigning it to one of the three categories ranging from 1 (inactive) to 3 (very active).

To assess the test-retest reliability, the questionnaire was re-administered to 21 mothers after one week; the correlations between test and retest for duration of low, moderate, and vigorous activities were 0.86 (p=0.01), 0.79 (p=0.04), and 0.94 (p=0.002) respectively. For the general question on overall activity, the level was 0.97 (p=0.001) .

### Statistical analysis

To compare total counts in boys and girls, between weekdays and weekends, the Mann-Whitney test was used. To estimate the degree of association between different days of measurements, the intraclass correlation coeficient (ICC) was calculated. Spearman correlation was used in evaluating the different ACC used. To evaluate the validity of the questionnaire, Spearman correlations were used for comparing percentages of different intensities of PA assessed from the questionnaire with the percentages of PA obtained from accelerometer cutoff points. To evaluate differences between minutes per hour spent in different intensities of PA, with the responses to the qualitative question by mothers, the Kruskal Wallis Test was performed. All tests were conducted with a two-tailed alpha level of 0.05. Analyses were performed using the SPSS for Windows (version 16.0).

## RESULTS

The mean age of the 35 children (51% girls) was 4.4 ±0.7 years (range 3-5 years), and the mean BMI was 15.8±2.7 kg/m^2^ (range 10.8-21.7). The average BMI was at the 65^th^ percentile for age and gender; 23% were either overweight or obese (BMI ≥85^th^ percentile for age and gender). Mean sleeping time was 9.8±1.1 h/day, and 33% of the children reported daily naps (0.7±1.2 h/day). The recorded mean time of accelerometer-use was 11±1.7 h/d for an average of 3.8±0.9 weekdays and 1.6±0.5 weekend days. ICC calculating the degree of association between different days of measurements was 0.76, 0.77, and 0.74 for school, weekdays, and weekends.

Total daily mean counts/15-s measured by accelerometer were 178±75: for weekdays 185±83, for weekends 152±65, and for the time spent at preschool centre 221±105.

The correlations between parents’ answers to the broad question assessing children's typical activity level and children's percentage of time spent in moderate (M), vigorous (V), and moderate+vigorous activity (MV) with ACC (using method of Sirard *et al.*) were r=0.57 (p=0.0001) and r=0.62 (p=0.0001) respectively (Table 1). Percentages of time spent in V and MV reported by parents on the questionnaire were significantly correlated with time spent in V from Sirard *et al.* [ACC r=0.53 (p=0.001)] and Pate *et al.* [ACC r=0.41 (p=0.01)] (Table 1). There were statistically significant differences between means in time spent in each PA intensity category (min/h) by ACC (Sirard *et al.*) and parents’ answers to the broad question about perception of their children's overall PA level (Table 2).

Table 3 shows the percentage of time spent in different PA intensities according to questionnaire responses and ActiGraph count cutoffs. It shows higher percentages of moderate to vigorous PA when using the Pate cutoff points. Additionally, similar percentages of sedentary PA at home were reported using the parents’ questionnaire and ACC cutoff points. Using the questionnaire, we observed differences between the percentage of PA at home and at school and the percentage of time spent in sedentary PA. No difference was found, using the Pate and Sirard cutoff points. Furthermore, parents overestimate time spent in vigorous PA (Table 3).

**Table 1.** Correlation coefficients between percentages of time spent in different PA intensities and perception of child's activity according to questionnaire responses and ActiGraph count cutoffs

Parents’ questionnaire	Accelerometer data (ACC)								
		ACC Sirard *et al.* 2005				Pate *et al.* 2006			
Physical activity		S	M	V	MV	S	M	V	MV
Sedentary (S)	Rho	0.35*	0.11	-0.40*	-0.50**	0.34*	0.05	-0.37*	-0.34*
Moderate (M)	Rho	-0.31	-0.23	0.40*	-0.13	-0.27	-0.07	0.34*	-0.04
Vigorous (V)	Rho	-0.62‡	0.40*	0.53†	0.54†	-0.45**	-0.02	0.41**	0.37*
M+V (MV)	Rho	-0.34*	0.31	0.61‡	0.49**	-0.13	0.03	0.43**	0.34*
Perceptions of child's activity	Rho	-0.45**	0.57†	0.50**	0.62‡	-0.35*	0.25	0.47**	0.35*

*=p<0.05;

**=p<0.01;

†=p<0.001;

‡p=0.0001

**Table 2.** Median differences in physical activity measured by ACC according to parents’ perception of children's PA

	Median physical activity [min/h^1^ (mean±SD)]			
	Sedentary (S)	Light (L)	Moderate (M)	Vigorous (V)
Perceptions of child's activity^2^				
Low	51.6 (52.5±3.0)	6.8 (6.1±2.5)	1.1 (0.9±0.5)	0.4 (0.4±0.2)
Moderate	51.6 (52.0±2.4)	6.1 (6.0±1.8)	1.5 (1.4±0.7)	0.7 (0.7±0.5)
Vigorous	48.0 (48.0±1.7)	7.9 (8.4±1.4)	2.5 (2.6±0.9)	0.9 (1.1±0.4)
p values^3^	0.007	0.02	0.004	0.017
Total	50.7 (51.3±2.9)	6.8 (6.4±2.0)	1.5 (1.5±0.9)	0.7 (0.7±0.5)

Low: normally sits down while playing, watching TV, colouring or playing with dolls or stuffed animals and singing. Moderate: combines playing while sitting down and standing up with activities that include walking from one side of the room or house to the other. Vigorous: does not stop moving, goes from one side of the house to the other, goes up and down the stairs, runs and jumps

ActiGraph count cutoffs, Sirard *et al.*, 2005;

By parents at home;

Kruskal Wallis Test;

SD=Standard deviation

**Table 3.** Mean percentages of time spent in different PA intensities according to questionnaire responses and ActiGraph count cutoffs

Reading source	% of time in sedentary PA		% of time in light PA		% of time in moderate PA		% of time in vigorous PA	
	Home	Pre-school centre	Home	Pre-school centre	Home	Pre-school centre	Home	Pre-school centre
Parent's questionnaire	83.0±8.9	-	-	-	6.0±4.7	-	11±7.2	-
Pate *et al*. 2006	87.4±4.2	85.1±5.6	-	-	9.0±2.9	10.6±4.0	3.5±1.6	4.3±2.0
Sirard *et al*. 2005	85.6±4.8	82.9±5.9	10.7±3.4	12.4±4.3	2.6±1.5	3.1±1.9	1.2±0.8	1.5±1.2

## DISCUSSION

This study shows that responses to the questionnaire on parents’ perceptions of their children's physical activity were moderately associated with the moderate and vigorous PA recorded with ACC. In addition, children who were shown by ACC to spend more minutes per hour on light, moderate and vigorous PA and less time in sedentary activities were more likely to be perceived by their parents as active (Table 2). This result indicates that the parents’ responses to the questionnaire provide a good estimate of preschool children's PA in this sample of children living in the northwestern region of Mexico. Other studies have shown similar results. Janz *et al.* reported an association between the responses to parents’ questionnaire on children's behavioural traits and vigorous PA. They also showed that children with the highest parental questionnaire scores were 2.7 times more likely to be in the highest percentile for total activity as measured by an accelerometer than those with low or moderate parental questionnaire scores (24).

In the present study, an association was found between the reported percentage of time spent in vigorous PA and moderate and vigorous PA from parents’ responses to the questionnaire and the results of ACC (using Sirard *et al.*). Likewise, Harro found a correlation in kindergarten and elementary school children between the duration of moderate and vigorous PA reported by the parents’ questionnaire and the Caltrac accelerometer (21).

In this study, we found that associations were higher, using the ACC by Sirard *et al*. (15) than when the Pate *et al.*'s ACC was used (25). Therefore, depending on the method used, the validation of a questionnaire or the assessment of the PA can yield different results. There is currently enormous variation in the practice between researchers and the use of cutoff points. In a recent review conducted by *Reilly et al.*, it was suggested that the biological plausibility should be considered, along with the quality of the evidence and the mass and consistency of the evidence to identify the best cuttoff points (29). Though based on different methods of deriving cutoff points, we have seen consistent results among 3-5 year old children, using cutoff points from Sirard *et al.* and Pate *et al*.

The children in this study were engaged (depending on the ACC used) from 2.8 to 8.8 min/h of moderate and vigorous PA at preschool centre (4.5-15% of the 4 hours recorded). This relatively low level of PA in the preschool children is consistent with the results observed in other studies conducted in different preschool settings (17,30). Pate *et al.* reported moderate and vigorous PA of 7.7 min/h (~13% of the time registered) (17). However, they found important differences among the preschool settings. Finn *et al*. also reported variation (30) in the time spent in vigorous PA depending on the centre that the children attended (3.6-6.0%). We did not assess differences between preschool settings.

During PA at home, 4-12% of time was spent in moderate and vigorous PA, slightly less than that measured in the preschool setting. The highest percentages of moderate and vigorous PA (Table 3) were recorded when using ACC by Pate *et al.* (25). As reported in other studies (31,32,33), the preschool children whom we studied did very little moderate and vigorous PA at home and were sedentary most of the time.

Our study also indicates that children at home and preschool centre, during weekdays and weekends, spend 80% of their time in sedentary behaviours (Table 3), which is less than the reported 90% as measured, using the same accelerometer methodology among Latino preschool children in Redwood City, California (9). The average BMI of participants in the California study was also higher (at the 85^th^ percentile for age and gender) than in Baja California (at the 65^th^ percentile for age and gender).

The questionnaire might be a simple and useful tool to assess the PA of preschool children at the Mexican daycare facilities, and the information collected might also be used for sending an alert for medical referrals, which will aid physicians in making a definite diagnosis and prescribing adequate treatment. Additionally, if validated in different settings, it could be used in promoting and evaluating preventive and intervention programmes.

Strength of the present study is the use of accelerometers to validate children's physical activity objectively. This study used the cutoff points reported by Sirad *et al.* (15), which were validated with direct observation. Additionally, cutoff points proposed by Pate *et al.* (25), using a metabolic criterion measure (VO_2_) for 3-5 years old children, were used. Therefore, the results of this study were evaluated using two different validated standards. This is the first study which validates the perceptions of Mexican parents about PA of their preschool children.

### Limitations

One of the limitations of our study is the use of uniaxial accelerometers. Uniaxial accelerometers worn at the waist do not measure upper-body activities or activities occurring in a horizontal plane (15). This might be relevant since movement patterns in young children might have higher horizontal motion than older children. Activities of 15 or more minutes were recorded in the questionnaire, and shorter activity periods were not reported. Activity patterns were tested at only one point in the year, which does not allow for quantifying seasonal variation, and only 10 hours of observation per day were recorded. Thus, children might have been more active or more sedentary than the results of the study indicate.

Additionally, this study was conducted using a convenient sample in one preschool centre in a city located in the northwestern part of Mexico; thus, we could not assess differences between children from different settings, or children with or without siblings. Results of this study may not be generalized to other settings, regions, cultural populations or socioeconomic groups within Mexico.

### Conclusions

Although parents’ assessments of their children's PA, as measured by the questionnaire, cannot be used to provide point estimates of their children's overall PA, these assessments were significantly correlated to accelerometers reading, a direct measure of physical activity. The questionnaire in this study might be a simple and useful tool to assess the PA of preschool children at Mexican daycare facilities. However, further investigation is warranted to assess if the results are applicable in other daycare settings.

**Appendix.** Physical activity questionnaire for parents (translated from Spanish)

Card number__________________ Boy () Girl ()

1. How much time per day does your child spend doing the following activities?
  Sitting down watching television. Sitting down playing (video games, toy cars or dolls, puzzles, colouring). Lying down in bed or an armchair.
  15 min. () 30 min. () 45 min. () 1 hour () more than 1 hour (how many hours?___)
2. How much time per day does your child spend doing the following activities?
  Walking to school. Walking to the park and in the park or on the patio. Walking in a mall or going to the convenient store (tiendita)
  15 min. () 30 min. () 45 min. () 1 hour () more than 1 hour (how many hours?___)
3. How much time per day does your child spend doing the following activities?
  Playing circle games. Playing ball. Playing and running. Playing on a bicycle or tricycle.
  Play-fighting
  15 min. () 30 min. () 45 min. () 1 hour () more than 1 hour (how many hours?___)
4. Does your child do any of these activities during the week?
  Karate, Gymnastics, Football, Ballet or jazz, Baseball, Swimming.
  _____ day(s) per week _____ hour(s) per day
5. How many hours does your child sleep at night?
  _____ hours
6. How many times a day does your child take a nap and for how long?
  _____ time(s) a day
  15 min. () 30 min. () 45 min. () 1 hour () more than 1 hour (how many hours?___)
7. Which of these phrases best describes your child's activity at home?
  - () Normally sits down while playing, watching TV, colouring or playing with dolls or stuffed animals
  - () Combines playing while sitting down and standing up with activities that include walking from one side of the room or house to the other.
  - () Does not stop moving, goes from one side of the house to the other, goes up and down stairs, runs and jumps.
